# Black/African American Breastfeeding Experience: Cultural, Sociological, and Health Dimensions Through an Equity Lens

**DOI:** 10.1089/bfm.2020.0312

**Published:** 2021-02-12

**Authors:** Adwoa Gyamfi, Barbara O'Neill, Wendy A. Henderson, Ruth Lucas

**Affiliations:** School of Nursing, University of Connecticut, Storrs, Connecticut, USA.

**Keywords:** African American, breastfeeding experience, equity

## Abstract

***Background:*** Disparities in breastfeeding (BF) continue to be a public health challenge, as currently only 42% of infants in the world and 25.6% of infants in the United States are exclusively breastfed for the first 6 months of life. In 2019, the infants least likely to be exclusively breastfed at 6 months are African Americans (AA) (17.2%).

***Materials and Methods:*** A scoping review of the literature was undertaken by using Arksey and O'Malley's six-stage framework to determine key themes of AA women's experience BF through an equity lens. Electronic databases of CINAHL and PubMed were searched for peer-reviewed, full-text articles written in the English language within the past 5 years by using the terms BF, AA, Black, sociological, cultural, equity, health, attitude, exposure, initiation, continuation, barriers, and facilitators.

***Results:*** Initially, 497 articles were identified, and 26 peer-reviewed articles met the eligibility criteria. Through an equity lens, three main themes emerged, which summarized AA women's BF experience: cultural (family, peers and community support; misconceptions; personal factors), sociological (prejudices, racism, home environment; financial status; sexuality issues; BF role models; employment policies), and health dimensions (family involvement; timely and honest information from staff; baby-friendly hospital initiatives; postnatal follow-up; special supplemental nutrition program for women, infants, and children).

***Conclusion:*** For AA women, exclusively BF is beset with diverse cultural, health, and sociological challenges. Multifaceted approaches are needed for successful resolution of BF challenges to bridge the racial gap in BF in the United States. Future studies may explore interventions targeted to modifiable barriers to improve BF outcomes.

## Introduction

Breastfeeding (BF) offers key preventative health benefits for women and their infants. To receive these optimal health benefits, exclusive breastfeeding must be maintained by women and their infants for the first 6 months of life.

The Healthy People 2030 (MICH-2030-15) initiative recognizes the health benefits of BF and has set a public health goal that 42.4% of infants in the United States be breastfed exclusively through 6 months.^1,2^ Although 42% of infants in the world meet the public health goal, only 25.6% of infants in the United States meet this target of being exclusively breast fed for the first 6 months of life.^1,2^ Even more concerning is that African American (AA) women in the United States,^3^ in particular, have the lowest BF initiation and continuation rates, with only 17.2% of infants exclusively breastfed at 6 months.^4^

The preventative health benefit of BF increases with longer BF duration. Women who report a cumulativ*e* BF history of 12 months or more decrease their risk for breast and ovarian cancers, diabetes, hypertension, and return to prepregnancy body shape.^3,5,6^ BF supports maternal well-being during the fourth trimester of pregnancy after birth by decreasing the risk of depression, anxiety, and stress symptoms, and it increases maternal self-efficacy.^7^

BF also provides lifelong infant health benefits; the longer infants exclusively breastfeed, the greater benefit in reducing infantile and respiratory diseases (COVID-19), sudden infant death syndrome, and chronic diseases related to obesity.^8–12^ Ultimately, the public health burden of not BF results in a global gross expenditure of U.S. $341.3 billion, with $114.97 billion incurred by the North America Region.^13^ The early cessation of BF (before 3 weeks) costs the United States an estimated $3.0 billion annually (2010 dollars) due to purchase of formula, increased infantile diseases and health visits, and lost wages ($2.3 billion being maternal costs).^13^

Many AA women encounter unique challenges in initiating and sustaining BF. Notably, inadequate social support,^4,12,14,15^ various forms of prejudices,^16^ racism,^17^ misconceptions about BF versus formula feeding,^18^ insufficient financial resources,^16,19,20^ as well as personal factors such as low self-efficacy, negative attitudes, unwillingness to breastfeed, misconceptions about the benefits,^21,22^ and inadequate resources.^23^

Support is a critical factor in addressing these challenges to meet and sustain BF practice.^4,15,24^ AA women who obtain some forms of support from relatives, health staff, and employers are more motivated to breastfeed.^15,25^ In addition, participating in programs such as Centering during Pregnancy, Baby-friendly Hospital Initiative (BFHI), and the “Supplemental Nutrition Program for Women, Infants and Children Services,” also known as the WIC Program, encourages BF initiation and duration. The aforementioned types of support contribute to women's BF self-esteem and it has been found that women with high self-esteem breastfed more frequently and for longer duration.^26–28^

The purpose of the scoping review was to explore the BF experiences of AA women, with critical attention to the cultural, sociological, and health dimensions through an equity lens to positively impact BF initiation and continuation in the United States.

## Materials and Methods

The scoping review was done based on the seminal scoping review method as outlined by Arksey and O'Malley.^29^ In the first stage, the research question to be addressed was identified as, “What is the experience of AA women in the United States?” In the second stage, articles were selected based on relevance to the topic from the databases of PubMed and CINHAL. The search terms adopted for the review were: BF, AA, Black, sociological, cultural, equity, health, attitude, exposure, initiation, continuation, barriers, and facilitators.

The third stage involved the selection of data. This was limited to full-text articles written in the English language from peer-reviewed journals, all research types (qualitative, quantitative, and mixed methods), and published within the past 5 years. Stage four concentrated on charting the data based on iterative process whereas stage five focused on collating, summarizing, and reporting the results. Lastly, for validation, a lactation consultant was asked to validate the scoping review analysis.

**FIG. 1.**
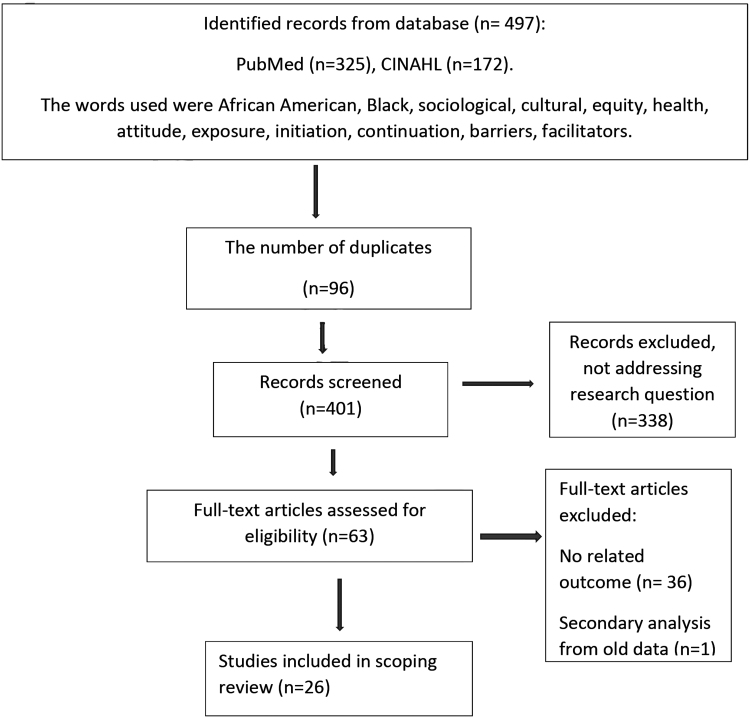
Flowchart of the article selection process for the scoping review.

The initial search of the electronic database resulted in the identification of 497 articles—with 96 duplicates, and 338 not addressing the research question. Subsequently, 63 full text-articles remained eligible for screening, 36 were excluded for unrelated outcomes, and 1 article was excluded that was a secondary analysis of historical data. Thus, 26 full-text articles remained for the scoping review analysis (Fig. 1). “Arksey and O'Malley framework” was used to conduct a quality assessment of the studies (Table 1).

**Table 1.** Scoping Review on Breastfeeding Initiation and Continuation Support Among African Americans

Author, year, study site	Method	Sample	Trustworthiness	Findings	Gap
Robinson et al., 2019, Southeast Wisconsin	Qualitative Two focus group discussions	9 AA women, ≥18 years currently BF or breastfed 6 months before the study.	Verbatim transcription, member checking, audit trail and expert support.	The role of BF peer counselors had a positive impact on lactation. Four themes: Educating with truth, validating for confidence, countering others negativity, supporting with solutions.	Establishment of standard guidelines for BF peer counselor's intervention.
Hinson et al., 2018, Northeastern US	Qualitative Six focus group discussions	United States born—AA woman, ≥18 years, within 3 months postpartum.	Prolong engagement, transcription, field notes, member checking.	BF initiative among AA is affected by: cultural beliefs, (benefits, bonding, natural, “wet nurse” history, formula is as good as breast milk, racism); sexuality issues (breasts for nutrition, over-sexualization of AA females, sexual act); social environment (familial/network influence—self, mother, sisters, partner, friends/peers, religious community); information sources (prenatal clinics, WIC, physicians, nurses, lactation consultants, internet), BF intentions (positive, negative or ambivalent), barriers (pain, embarrassment of public exposure, lack of knowledge and support of BF in AA community, lack of information and education about BF, prenatally lack of access to equipment and resources, aversions to BF, convenience of bottle and FF independence, national policy), and facilitators (involved fathers, prior positive experience, cost and convenience of BF, peer counselors/peer support groups, supportive family environment, BFHI, WIC).	Multifactorial approach, involving mothers, partners, and community to address cultural barriers to lactation.
Schindler-Ruwisch et al., 2019, Washington, DC	Qualitative 24 Interviews	24 AA women aged ≥18 years, in WIC with infants 0–8 months.	Prolong engagement, transcription, dual coding, Kappa 0.7107.	Four themes of BF initiation: Influence of others on BF confidence and intention; benefits; pervasiveness of obstacles (pain, latch, milk supply); and importance of social support (emotional, instrumental, appraisal, informational, provider support WIC).	Reinforcement of community outreach program through social support geared toward BF.
Furman et al., 2016, Cleveland	Quantitative Pre- and post-test	66 Partners/partners, predominantly self-identified AA, aged 17–64 years.	No validity or reliability tests done.	Improved knowledge in lactation (62%) and readiness to welcome BF of next infant (85%).	Engagement of inner city and AA fathers or partners as priority in BF initiatives.
Oniwon et al., 2016, Washington, DC	Quantitative Cross-sectional	100 multiparous women in WIC, predominantly AA, aged 18–41 years.	No validity or reliability tests stated.	Women (71%) initiated BF based on education level, full-time or no job status, had one child, and was breastfed in childhood. Barriers for BF continuation at home—limited support, pain, insufficient milk perception.	Improvement in prenatal education, and reinforcement of BF exclusivity in though outpatient services.
Barbosa et al., 2017, Richmond, VA	Qualitative Seven focus group discussions	25 low-income AA women, aged ≥18 years, enrolled in WIC, with a child <2 years.	Transcription, member checking.	Positive deviance factors—BF intention, high self-efficacy and sought knowledge, value BF and understood FF disadvantage, varied interpersonal support, resist negative BF/positive FF influences, positive BF influence on others, prenatal health providers promote BF and FF products, hospital offered good BF instruction and support, postnatal staff gave limited support/advise, appreciate WIC BF help, overcame job barriers, indifferent to BF stigma and public BF.	Community engagement to promote strengths of positive deviance toward BF.
Asiodu et al., 2017, Northern California	Qualitative Ethnographic	22 AA (14 pregnant women and eight support people).	Prolong engagement triangulation	Themes related to BF decision making—best for baby; normalization/role models; social support; fluid social dynamics, resiliency; seeking support and empowerment; stress, shame, and guilt; combination feeding.	Education through identified social support person, social media platform, and combination feeding.
Thomson et al., 2017, Mississippi	Quantitative Experimental	82 AA	Randomization No blinding High attrition rate.	BF knowledge increased in both groups, 39% participants BF infant for ≥6 months. Pre-pregnancy weight and BF intent were significant for BF initiation.	Using peer counselors with BF experience and inclusion of male partners in education.
Robinson et al., 2018	Quantitative Systematic review and meta-analysis	Four identified	PRISMA guidelines	AA pregnant women who participated in Centering Pregnancy Models had a 71% probability of BF observed.	Outcomes of Centering Pregnancy model for AA women
Merewood et al., 2019, Southern US	Quantitative Longitudinal (2014–2017)	Hospitals enrolled in Communities and Hospitals Advancing Maternity Practices	Validity and reliability tests not stated. No blinding	BF initiation and exclusivity among AA infants increased from 46% to 63% (*p* < 0.05) and from 19% to 31% (*p* < 0.05), respectively.	None stated.
Kim et al., 2017, Illinois	Mixed method Interviews and questionnaire	15 first-time AA mothers enrolled in WIC.	Validated instrument used (IIFAS and BFSE-SF) data saturation reached, transcription, member checking.	BF facilitators and barriers—Themes: Normative infant behavior in sociocultural context; cultural beliefs on maternal nutrition and BF; time and cost associated with BF; managing and integrating BF while maintaining a social life; necessity of social support from significant others and female role models; suboptimal support from institutions (hospitals, school, work place and community). Positive attitude to BF (70%) and high self-efficacy (62%).	Interventions focusing on social support (emotional, tangible, informational, and encouragement).
Robinson et al., 2019, Internet	Qualitative Four focus group discussions	22 AA women, aged ≥18 years, participating in BF support groups on Facebook.	Transcription, peer debriefing, member checking, expert input, triangulation.	Themes: Creating a community for Black mothers; online interactions and level of engagement; empowerment of self and others; shifts in BF perceptions and beliefs.	None stated
Robinson et al., 2019, Online	Quantitative Online cross-sectional survey	277 AA women	Validated instruments used (IIFAS and BFSE-SF)	Average BF intention duration was 19 months, due to Facebook support. Self-efficacy and BF attitudes remained significant predictors of intended BF duration.	Impact of Facebook support on prolonged BF durations in AA women.
Spencer et al., 2015	Qualitative Sequential-Consensual Qualitative Design	Stage 1: Four AA key informants Stage 2: 17 AA mothers BF infant for ≥4weeks Stage 3: 7 AA women	Prolonged engagement, transcription, peer debriefing	Stage 2 themes: Self-determination; spirituality and BF; and empowerment Stage 3 themes: Engaging spheres of influence; sparking BF; activism; and addressing images of the sexual breast versus the nurturing breast.	Engaging supportive network; pediatricians' and obstetricians' view on BF attitude and knowledge; culturally sensitive educational interventions and initiatives (mother's time, activities).
Obeng et al., 2015, Midwest	Qualitative Focus group discussion	20 AA women, aged 20–40 years	Prolonged engagement, verbatim transcription	Themes identified on AA women BF perceptions and experiences: Health benefits, lack of information, negative perceptions of BF by others, organizational support, unforeseen circumstances of BF.	Explore ways to increase BF initiation and duration among AA women based on their experiences, desires, and needs.
Johnson et al., 2016, Detroit	Qualitative Focus group discussion	38 Pregnant and lactating AA women and racial diverse health professionals	Transcription,	Health workers not always supportive of BF, lacked adequate information and skill to educate AA women on BF, so women lost confidence in them and relied more on relatives and peers.	None stated
Jefferson, 2017	Quantitative Cross-sectional survey	696 AA and Caucasian college students, aged ≤45	Validated instrument used, IIFAS	Favorable attitude to BF but FF was viewed as much easier; odds of experiencing BF exposure and positive BF attitudes were thrice higher for Caucasian students than for AA students.	Further studies to identify strategies to improve BF exposure and attitudes among AA students.
DeVane-Johnson et al., 2017, Online	Qualitative Integrative literature review	Four social science electronic databases, 47 peer-reviewed articles.	Theme validation by two independent authors	Themes for BF disparities among AA women: Social characteristics (e.g., low socioeconomic status, single); perceptions of BF; quality of BF information provided by health care providers.	Focus on sociohistorical factors that have shaped current norms of BF among AA women.
Kamoun and Spatz, 2018, West Philadelphia	Mixed methods Interview Survey	Interview: 10 leaders, 44 members and 11 leaders surveyed, ≤45-year, AA, Muslim, both sexes.	Validated instrument used, IIFAS; verbatim transcription, member checking.	No prevalence of Islamic education on BF; favorable views about BF, attitude toward BF improved through religious incorporation on BF education.	None stated
Moon et al., 2017	Quantitative Randomized controlled trial	1,194 AA women who had just delivered, aged 18–42 years.	Random selection and assignment	BF was 5.3 and 6.1 weeks for infants who room or bed-shared (*p* = 0.01). Exclusive BF was 3.0 and 1.6 weeks for infants bed-shared or room shared (*p* < 0.001). Group assignment did not affect BF duration. AA infants <6 months were mostly room shared.	Further research on factors that improve BF exclusivity and duration.
Lutenbacher et al., 2016	Qualitative Focused group discussion	16 Self-identified AA women with one birth history within 5 years.	Prolonged engagement, verbatim transcription, field notes, peer debriefing.	Factors affect BF: four themes—Balancing the influences of people, myths, and technology; being in the know; critical periods; and, supportive transitions.	None stated
Fabiyi et al., 2016, Central Ohio	Qualitative Interviews	20 Middle-class AA and African-born women	Verbatim transcript, prolonged engagement, member checking	Factors affecting BF: Persistent support and encouragement; dissuasive remarks; challenges with health, job, lactation, ambivalent BF attitude.	None stated
Deubel et al., 2019, Florida	Qualitative Interviews	20 AA women	Prolonged engagement, verbatim transcription.	BF challenges—No maternity leave, access to electric pumps, BF role models and or support network to normalize long-term BF, social pressures to initiate formula supplementation, fears that BF renders infants overly dependent on mother's care.	Monitoring the practical implementation of insurance coverage for BF pumps on rates of BF initiation and duration.
LoVerde et., 2018	Qualitative Interviews	Nine AA women with VLBW (≤1,500 g)	Prolonged engagement, point of saturation, member checking, verbatim transcription	Facilitators to BF: Being a mother; neonatal intensive care unit environment; community support; useful resources. Barriers: Maternal illness; milk expression; challenging home environment; emotional distress.	Perceived barriers to improve provision of mother's own milk and quality of lactation journey of AA women with preterm infants.
Robinson et al., 2019	Mixed methods Scoping review	MEDLINE articles (5) using PubMed, CINAHL, Cochrane Library, PsycINFO, and Sociological abstracts	Preferred Reporting Items for Systematic Reviews and Meta-Analysis (PRISMA) guidelines.	AA women experience racism, bias, and discrimination affecting BF care, support, and outcomes.	Effect of racism, bias, and discrimination on BF care, support, and outcomes.
Griswold et al., 2019	Quantitative Secondary data analysis of Black Women's Health Study (1995–2005)	2,705 for BF initiation analysis, 2,172 for BF duration analysis.	Validity and reliability tests not stated.	Racism in work environments was associated with lower odds of BF duration at 3–5 months; whereas higher odds of BF initiation duration at 3–5 and 6 months were observed in study participants who had experienced racism with the police; U.S.-born AA or having one parent born in the U.S. predicted lower odds of BF initiation and duration; residence in segregated neighborhood (mainly Black residents) during childhood decreased BF initiation and duration as compared with living in predominantly White communities of residence.	Influence of different racism experiences on BF behaviors for Black women in the United States and innovative interventions.

Scoping Review Chart on Quality Assessment of Studies as Guided by the “Arksey and O'Malley Framework.”

AA, African American; BF, Breastfeeding; BFHI, Baby-Friendly Hospital Initiative; BFSE-SF, Breastfeeding Self-Efficacy Short Form; FF, formula feeding; IIFAS, Iowa Infant Feeding Attitude Scale; VLBW, very low birth-weight infants; WIC, Special Supplemental Nutrition Program for Women, Infants and Children.

## Results

Initially, 497 articles were identified, and 26 peer-reviewed articles met the eligibility criteria (Fig. 1). Table 1 provides the complete overview of findings from each of the reviewed articles. Through an equity lens, three general themes emerged to describe the BF experiences of AA women in the United States, namely, cultural, sociological, and health dimensions. The definitions of the themes cover three dimensions and include: (1) Cultural, which includes the personal and familial network values influencing or inhibiting women to breastfed; (2) Sociological, which includes the larger community perception of BF, sexualization of the breast, and the societal issues of prejudice and racism toward AA women BF; and (3) Health, the interface between women and their health care providers, and health care system to support BF.

### Theme one: Cultural dimension

Culture defined the BF decisions of AA women in the United States.^16,22,30^ The family, peers, and community were important agents for social support. Families, peers, and community persistent support and encouragement for women boosted BF.^19,31–33^ The communal networks were instrumental for education, counseling, appraisal, interaction, engagement, successful transitions, positive deviance, reinforcement, and emotional well-being.^26,34,35^ The AA women who breastfeed exhibit positive deviance. Positive deviance refers to the fact that such women chose to breastfeed their infants contrary to the cultural norms of not breastfeeding post-slavery. Such influences became possible through in-person interactions, virtual platforms, and religious affiliations.^30,33,36^

Various forms of misconceptions on BF were observed within the AA community. For instance, some relatives, peers, and close neighbors claimed that breastfed infants become overly dependent on their mother; such myths pressurized women to initiate formula supplementation.^23^ Formula supplementation was seen as inexpensive, required less time, and allowed women to manage and maintain their social life.^20,37^ Conversely, social media engagement brought shifts in maternal BF beliefs and perceptions,^38^ which resulted in increased willingness to breastfeed beyond the infant's first year.^36^

Personal factors influenced the BF experiences of women. Maternal self-determination, positive attitude, positive deviance, high self-efficacy, spirituality, and empowerment^20,32^ motivated women to breastfeed. On the contrary, women who experienced stress, shame, guilt,^34^ embarrassment of public exposure,^16^ prejudiced public perceptions, challenges with milk expression,^21,22^ BF pain, and previous limited BF success^39^ were more likely to not breastfeed compared with the other women.^26^

### Theme two: Sociological dimension

Issues of prejudice and racism may have an influence on AA women's BF practices.^32,39^ Health professionals, for example, pediatricians and obstetricians, negatively posited that AA women are less likely to breastfeed their infants and had less BF knowledge.^17,32,39^ Prejudice in BF is grounded in historical antecedents, where AA women were mandatory “wet nurses”^16^ to the slave masters' children instead of BF their own infants and current racial challenges.^18^

For instance, racism in the workplace was associated with lower odds of BF duration at 3–5 months; whereas higher odds of BF duration at 3–6 months were observed in study participants who had experienced racism with the police. In addition, lower odds of BF initiation were reported in U.S.-born AA women or a woman with a U.S.-born parent and residents of mainly Black communities as compared with women who lived in predominantly White communities in childhood.^18^

Household composition and living arrangement are critical components of the social life of AAs.^16^ AA women mostly lived in multigenerational households or as single parents.^16,40^ The home environment affected maternal BF decisions and support postpartum.^25^ For instance, women who lived in resource-limited communities experienced major financial challenges.^16,20,40^ Therefore, the limited income constrained maternal access to the procurement of electric BF pumps and additional BF resources.^16^ Such experiences influenced the perceptions of some of the women to view BF as expensive.^16^

Working AA women, in particular, indicated a persistent need for organizational support toward BF.^19,39^ Support recommendations included paid maternity leave, absence of dissuasive remarks, encouragement toward maternal BF efforts, access to electric pumps, and insurance coverage for BF pumps.^19,23^ Women were optimistic that addressing these factors will overcome stigma around public BF.^26^ Moreover, the need to promote national policies favorable to BF at the workplace was recommended.^16^

The over-sexualization of AA women's breast was an issue, specifically emphasis of the breast for sexual acts rather than nutrition.^16^ Thus, suggestions were made to engage all spheres of influence to address images of the sexual breast versus the nurturing breast.^32^ In addition, BF role models were noted to be important in the BF experience of AAs.^20,23,41^ Such role models focused on emotional, tangible, informational, and encouragement interventions for women. Older sisters and grandmothers were recognized as the best suited for such roles.^20,23,41^

### Theme three: Health dimension

The health dimension theme encompassed the importance and value of a supportive health care system and supportive health care professionals toward the success of women's BF experiences. For instance, timely and honest information from staff, WIC, BFHI, postnatal support, and follow-up was identified.^16,19,21,22,32,39,40,42^ Such information was meant to promote persistent support and encouragement, which included training on milk expression.^21,26^ The quality of BF information provided by health care providers was important.^40^ Thus, culturally sensitive educational interventions and initiatives responsive to women's time and activities were stressed.^32^

When health care workers failed to include such interventions, staff were deemed not supportive and lacked adequate information and skill to educate AA women on BF. As a result, women lost confidence and relied more on relatives and peers.^42^ In addition, women preferred a system and professional approach to be inclusive of the partners in BF decisions.^25^

Another health care system mentioned by women was the role of peer counselors and there was an indication of a positive impact on lactation.^38^ Women viewed peer counselors' educational efforts as truthful, confidential, supportive, and helped dispel misconceptions about BF. Although effective, there remains a need to establish standard guidelines for peer counselors' BF interventions.^38^ An exemplar of the integration of the community, health care system, and BF is the Communities and Hospitals Advancing Maternity Practices Program.^28^ AA women received community-based perinatal BF support, which contributed to increased BF initiation (46–63% [*p* < 0.05]) and exclusivity (19–31% [*p* < 0.05])^28^ rates.

## Discussion

This scoping review provides valuable insight into the BF experiences of AA women in the United States. Through an equity lens, three main themes were identified that influenced the BF experiences of AA women in the United States, namely, the cultural, sociological, and health dimensions.

Similar to earlier findings, the cultural experiences of racism, positive deviance, personal factors such as self-esteem, maternal attitude, and BF misconceptions^16–18^ contribute to AA women's BF outcomes. These findings suggest that AA women need support with the mitigation of existing misconceptions and racial challenges to bridge the gap in BF. Such efforts may be achieved through the strengthening available via social support efforts by the familial associations, social networks.^19,31–33,36^

In addition, active inclusion of religious bodies as primary partners in BF promotion with the AA population should be included as part of cultural and sociological targeted interventions.^19,31–33,36^ Exemplars for these community-, state-, and tribal-level interventions targeting BF promotion for all women of diversity are supported by the national coalition of organizations, the United States Breastfeeding Committee (USBC). The USBC is committed to mitigating barriers by addressing the essential components of culturally competent BF care: consider Culture, show Respect, Assess/Affirm differences, show Sensitivity and Self-awareness, and do it all with Humility (CRASH).^43^

AA women's need for socioeconomic support was confirmed in this review.^16,19,20^ A significant social barrier in AA women's BF is the lack of BF role models across generations.^23,41^ An additional barrier is the perceived social value of the female breast as a sexual organ and not a source of nutrition.^16^ Targeted messaging is needed to promote the nutritional value and less emphasis on the over-sexualization of the AA female breast. Together, the change in messaging will support women to feel more comfortable to breastfeed with less stigma and better self and public acceptance.

Lastly, as observed in previous studies, women called for health care systems and health care providers to support their BF efforts.^15,25^ In this scoping review, women emphasized the need for persistent, truthful, family-inclusive, culturally sensitive support from the first to the fourth trimester.^28,32,42^ Thus, health professionals who actively engage with AA women throughout the four trimesters of pregnancy must proactively promote BF initiation and continuation. Further, BF support needs to integrate perinatal programs beginning in the community and continuing to the hospital setting and then back into the community so women and infants can receive the benefits of BF.

## Conclusions

AA women's BF experiences are confronted with diverse and unique challenges. These challenges require the collaborative efforts on the part of the individual woman, her family and peers, her community (religious institutions and employers), the health care system and its providers, as well as national policies for successful mitigation. The results of such efforts will address the current gap in BF initiation and continuation of BF for AA women and infants in the United States. Future studies should explore social support, including the role of the religious community, and its influence on AA's BF outcomes.
